# Bats of a Gender Flock Together: Sexual Segregation in a Subtropical Bat

**DOI:** 10.1371/journal.pone.0054987

**Published:** 2013-02-18

**Authors:** Eran Levin, Uri Roll, Amit Dolev, Yoram Yom-Tov, Noga Kronfeld-Shcor

**Affiliations:** 1 Department of Zoology, Tel Aviv University, Tel-Aviv, Israel; 2 Israel Nature and Parks Authority (INPA), Megido, Israel; University of Regina, Canada

## Abstract

Competition has long been assumed to be a major driver in regulating ecological communities. Intra-specific competition is considered to be maximal as members of the same species use the same ecological niches in a similar way. Many species of animals exhibit great physiological, behavioral, and morphological differences between sexes (sexual dimorphism). Here we report an extreme geographical segregation between the sexes in the greater mouse-tailed bat (*Rhinopoma microphyllum)*. To gain insight into the driving mechanisms of sexual segregation outside the mating season, we collected and integrated environmental, behavioral, physiological, and spatial information. We found that both sexes choose roosts with similar characteristics and the same food type, but use different habitats for different durations. Males forage around cliffs at higher and cooler elevations while females forage in lowlands around a river delta. We suggest that it is their different physiological and social needs, and not competition, that drives sexual segregation in this species.

## Introduction

Intra-specific competition is considered to be the most intense form of competition, as individuals of the same species are both similar in their biology and use similar habitats in a similar fashion [1]. Males and females of the same species may differ, however, in several aspects of their biology (e.g., sexual dimorphism, different thermoregulatory strategies), which may result in differences in use of the ecological niche (sexual segregation, [2]) and/or decreased competition between the sexes [3], [4].

Sexual segregation and sexually-derived character displacement have been studied in many vertebrate species, mainly ungulates and carnivores, but also in birds and bats [3], [5], [6], [7], [8], [9], [10]. Two major hypotheses, not mutually exclusive, have been posited to explain sexual segregation. One is that it reduces competition between the two sexes through the exploitation of different food sources, habitats, or activity times [9]. The other suggests that various unique innate biological differences between the sexes (which may result from sexual selection, reproductive role, etc.) could cause them to prefer different niches [11], [12]. Such differences include hormone levels, lactation, water balance, exercise capacity, adipose to muscle tissue ratio, body size, sociality etc., which, in turn, may influence thermoregulation patterns [13], inclination to aggregation [14], or differences in geographic dispersal patterns between the sexes [15]. These differences should be more pronounced when mammalian females are gestating or lactating [16].

Bats are particularly interesting in the context of sexual segregation, since sexual dimorphism in bats is rare but sexual segregation is widspread [17]. Seasonal sexual segregation has been documented in many bat species [18], [19], [20], [21], [22], [23], [24]. These include latitudinal sexual segregation, documented mainly in temperate-zone bats, in which males tend to winter at higher latitudes than females (reviewed by [24]); and altitudinal sexual segregation, in which males tend to forage at higher altitudes than females [17].

Many bat species form large colonies during at least part of their annual cycle. The evolutionary forces leading to the formation of mixed colonies or maternity colonies in bats have received considerable attention. However, the forces behind the formation of male colonies have rarely been studied [19], [20]. Safi and Kerth [25] hypothesize that the formation of male colonies relates to information-transfer, and proposed two factors that may be conductive to information-transfer-based sociality in bats: 1) bats that feed on patchy and swarming prey will forage in groups and transfer information about the location of prey; and 2) bats with high aspect-ratio wings will forage in low cluttered habitats, over a large-scale area, employing high intensity echolocation calls. Such bats will benefit from foraging in a group, which will facilitate efficient scanning of space and transfer of information via echolocation signals [26], [27].

To further explore the mechanisms driving latitudinal and altitudinal sexual segregation in bats, we studied the morphology, diet, foraging sites, and roosting sites of the greater mouse-tailed bat (*Rhinopoma microphyllum*). *R. microphyllum* is a medium-sized subtropical bat (25 gr) with high aspect-ratio wings, which forms sexually-segregated colonies composed of several thousand individuals in northern Israel during the summer (June to September) [28], [29], [30], [31]. During this period both sexes feed mainly on the swarming alates of the carpenter ant *Camponotus felah* [29], [30], which is common throughout the Mediterranean region of Israel. It performs one diurnal nuptial flight in April, and nocturnal nuptial flights every night during June to October [29], [32]. We tested the two major hypotheses explaining latitudinal/altitudinal sexual segregation: reduced competition and different habitat preferences of the two sexes.

## Materials and Methods

### Study area

The Hula Valley and the Sea of Galilee Valley are both parts of the Jordan River Valley, which is part of the Great Rift Valley. The Golan Heights, a volcanic mountain ridge, constitute the eastern border of the valley, and the limestone Ramim mountain ridge is its western border. The vegetation on the eastern slopes of the valley features mainly annual cereals in a savanna-like landscape of *Ziziphus* trees and shrubs (*Z. spinachristi, Z. lotus*) and oak trees (*Quercus ithaburensis*). The eastern slopes of the valley are rocky, partly covered with Mediterranean groves of *Quercus calliprinos*, *Pistacia atlantica* and *Pistacia palaestina* and planted forests of *Pinus halepensis*. The valley itself is covered with orchards and cultivated land. The Jordan River connects the Hula Valley (150 m above sea level) with the Sea of Galilee Valley (−200 m below sea level) 10 km to the south.

### Bat roosts, body mass and wing area

**Figure 1:**
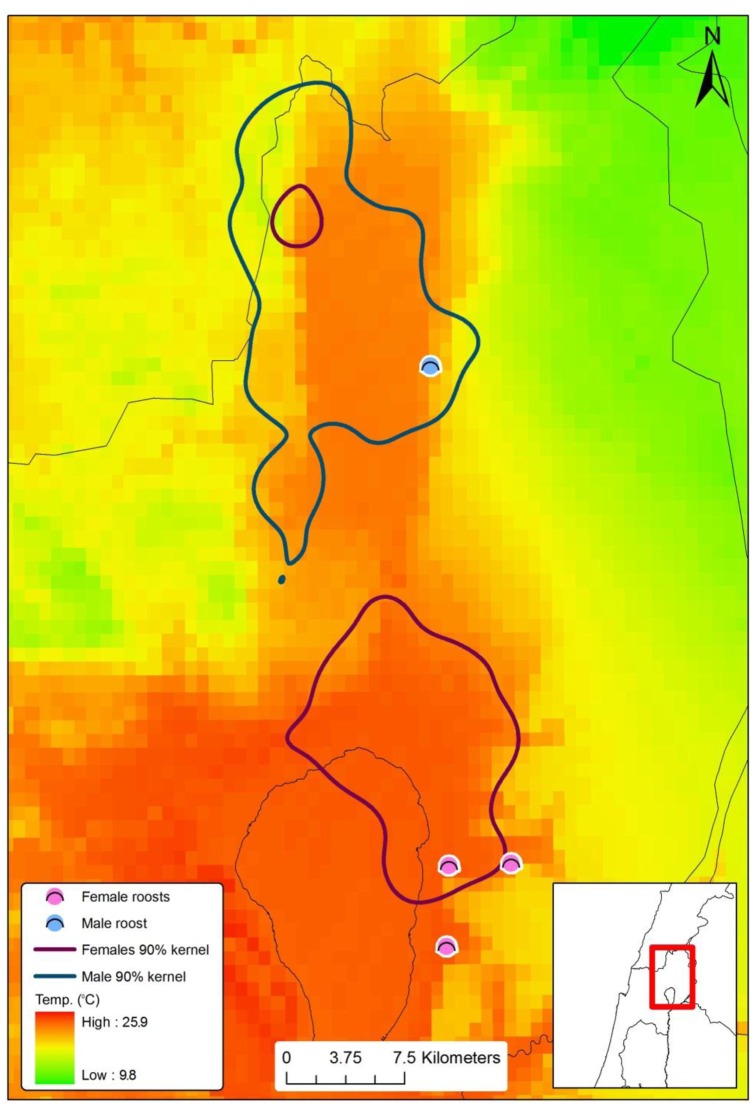
Male and female summer roosting locations, 90% kernel and roaming regions (blue  =  males' red  =  females) against a summer minimum temperature background. The small female foraging site in the middle of the males' region represents only one female – Da (see results).

Summer roosting caves of the bats (Figure 1) were monitored continuously between the years 2003–2011. During this period, 425 adult males and 402 adult females were observed, caught in the roost with hand-nets during the day, marked (alloy bat ring 4.2 mm, Porzana UK), and their body mass measured using an electronic balance (0.1gr). Fifty-one bats (28 males and 23 females) were photographed with spread right wing on a millimetric scale, and the digital images were later analyzed for area and aspect ratio using BATWING software. During this period the two sexes occupy different roosting sites. Female bat maternity caves are located around the Sea of Galilee (32°50’N 35°35’E, 200 m b.s.l), while the only known two male day-roosts lie about 40 km to the north (33°05’N 35°35’E 150 m a.s.l). The largest male colony (3,000–5,000 individuals) is located in a cave on the lower slopes of the Golan Heights, slightly east of the Hula Valley, and is inhabited from late May to early September. The other known male colony is located on the western side of the valley (the Naftali Mountain Range). *R. microphyllum* inhabits this cave only from mid-July to August and in relatively low numbers (500–1,000 individuals). This is the northernmost edge of the world distribution of this species.

### Radio-telemetry

During the summers of 2007 (23 Jul-10 Aug) and 2008 (26 Jun-1Aug), a total of 38 *R. microphyllum* were tagged with radio-transmitters (19 females, 19 males). In 2007 we tagged 10 males and 10 lactating females. In 2008 we tagged 10 males and, since no reproductive females were observed in any of the *R. microphyllum* colonies in Israel during that summer, we tagged nine non-lactating females. We used BD2-CT radio transmitters (Holohil Systems, Canada, 0.9 g for females and 1.1 g for males). Every night of the study, two observers located the bats using R-1000 receivers (Communications specialist INC. USA), and three-element Yagi antennas. We followed the males outside their cave for two consecutive nights; then tracked the females for two nights, continuing for the three-week lifespan of the transmitters. While bats were in motion (mainly during the first hour) we sought to capture an individual’s location as frequently as possible, when bats were foraging and remained in the same area we captured their locations every 30 minutes.

#### Data analysis

ANCOVA was used for comparing mass gain of males and females during summer. Average body mass and wing load of males and females was compared using a t-test. Results are presented as averages ± SD. For spatial analysis, we only used locations taken from a distance of 0.1–3 km. All bat localities were uploaded to a geographic information system (ESRI Arc View ver.9.31) for analysis. We used all female or male locations to compute kernel densities from 10–90% in 10% increments. We then divided the locations of the bats into those occurring in the first hour after emergence and those occurring later, and repeated the kernel analysis. We determined the habitat preferences of the bats using a geographically weighted regression (GWR) on their localities [33]. We divided the foraging areas into grid cells of one km^2^, encompassing 23*28 km for either males or females. Each cell was valued by summing the number of bat occurrences in it (this approach also minimized possible triangulation errors). We evaluated the effects of different spatial prediction parameters on the occurrence of the bats.

For each cell we calculated the values of: 1) Average daily temperature in August [34]; 2) Average annual rainfall [34]; 3) Differences in elevation between highest and lowest point in a cell; 4) Standard deviation of elevations within a cell; 5) Habitat type: the study area was divided into polygons of eight habitats: agricultural fields, orchards, Eucalyptus stands, natural forests, wetland/water habitats and human settlements. If a cell encompassed more than 50% of a specific habitat it was defined as such. If a cell comprised less than 50% of any habitat it was defined as a mixed habitat; 6) A cell that encompassed more than 20% of human settlements was defined as settlement (binary variable); and 7) A cell that included a water source was also defined as holding water (binary variable). The last two variables (settlements and water) were considered to exert a strong peripheral impact and so were deemed as more influential. In all models bat localities were used as the dependent variable and different combinations of explanatory variables (see above) were tested. We used the values of the AICc weights to rank the best model or model sets. Each analysis was conducted for the first hour localities (commuting flight to foraging sites) and then for localities measured afterwards (foraging).

Captures and radio tagging of bats were carried out under license of the Israeli Nature and Parks Authority (NPA), licenses number 2003/1732, 2004/18248, 2005/21980, 2006/25057, 2007/30126, 2008/31142, 2009/33059, 2010/37905.

## Results

The two largest *R. microphyllum* colonies consisted of 3,000–5,000 bats each, throughout the summer period. During the last nine years of the study we surveyed this specific study area, and found more than 50 potential roosts for *R. microphyllum* (dry and shallow caves). Nevertheless, we found *R. microphyllum* only in certain caves and abandoned man-made structures. When disturbed by fire (mainly on the dry grassy lands in the eastern part of the valley) or by intensive human activity, the bats changed their roost as a group, but later returned to their original roost when the disturbance ended. No males or females were detected in the opposite sex’s roost until the end of summer (with one exception, see below). At the end of August males left their roost and were occasionally observed in the southern female roosts between September and October. These males were not reproductively active (*R. microphyllum* develop testes and mate in April). Females gave birth at the beginning of July. Females and juveniles began to leave their roosts from early September, and all summer roosts were abandoned by late October.

**Figure 2:**
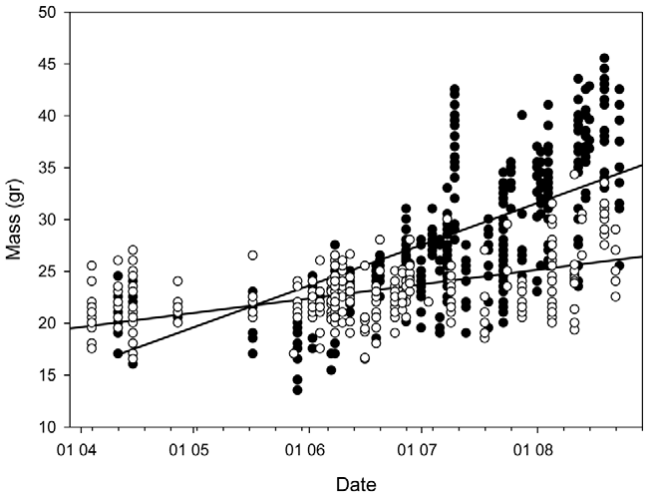
Male (empty circles) and female (filled circles) *R. microphyllum* body mass during the summer. (slope 0.19 and 0.06 for males and females respectively, ANCOVA: P<0.0001, F = 247).

During the first 20 days spent at the summer roosts there was no significant differences between male and female body mass (t-test: t = 1.21 d.f.  = 166, P = 0.225). However, body mass gain during summer differed between male and female *R. microphyllum*, with males gaining body mass more rapidly than females (Figure 2, significant difference between the slopes of the two sexes, ANCOVA: F = 247, d.f. = 824, P<0.0001,). We found no significant difference between the sexes in their wing area size: males: n = 26 142±19 cm^2^; females: n = 24, 149±23 cm^2^ (t-test: t = 1.2, d.f. = 48 P = 0.4).

Both sexes left their roost at dusk and flew in large groups, several hundred meters above ground, to their foraging sites. Foraging areas of both sexes were about 12 km distant from their roost. We were able to detect the bats using a directional antenna from a distance of up to 10 km (bats could be detected from the other side of the valley upon their evening emergence), but triangulation locations were taken from much shorter distances (0.1–3 km) to reduce triangulation error. In a few cases we obtained visual and audio contact (we were able to receive radio signals without antenna, and hear *R. microphyllum*'s low harmonic echolocation call), of foraging bats that were flying in large groups about 20 m above ground. We succeeded in tracking all the 38 radio-tagged bats during their foraging bouts (average 10±6 and 12±6.6 positions for each male and female, respectively, see Table S1, supplementary data), although about 50% of the bats changed their day roost after being fitted with a radio transmitter.

During the first hour after leaving the roost the main activity of males took place around wetlands in the Hula valley (n = 42). The best model that explains the spatial dispersal of male bats in the first hour of foraging is that of proximity to wetlands, explaining 32% of the spatial variation in their dispersal (see supplementary data, Table S2, for model results). This was surprising, since in nine years of intensive work with *R. microphyllum*, both in the wild and in the laboratory, they had never been observed drinking water and they do not consume any aquatic insects (see [29]). Moreover, unlike many other open-space foraging bat species, *R. microphyllum* was never caught in mist nets positioned above water in more than 30 years of bat surveys in Israel.

**Figure 3:**
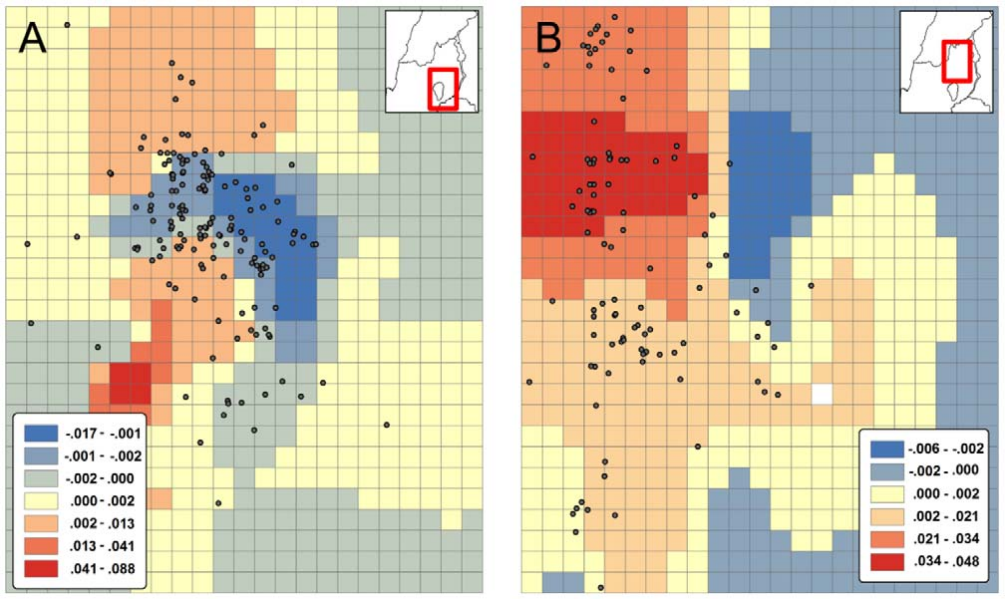
Female (A) and male (B) GWR model coefficients. For females the best predicting model included only altitude span for each cell. For males the best predicting model included only the altitude standard-deviation for each cell. Also shown are the localities of the bats (upper right corner) in the time frame of the analysis – after the first hour of activity.

After the first hour of nocturnal activity the males moved west to the far western side of the Hula Valley, close to the Naftali mountain range, about 12 km from their roost. The best model that explains their activity during this period is related to maximum altitude standard deviations, with males seeking those cells with greatest differences (Figure 3B). The radio-tagged bats were often recorded aggregated at a particular large cliff in that area. On one of the nights in summer 2008 we recorded all nine males together at the same spot on this large cliff.

During summer nights, after emergence, females flew directly to the Jordan River delta (Bethsaida valley) – about 12 km from their roosts. For females the best model explaining their occurrence during both parts of the night was altitude differences as a sole predictor. Most females sought flat areas – with minimal altitude differences within a cell (Figure 3A). The minimum ambient temperature in the foraging ranges of the two sexes (in both the 90% and the 10% kernel densities) was about 3°C higher in the females’ foraging range (18.9°C vs. 22°C minimum during summer).

One non-reproductive female (Da), displayed different behavior from all the other individuals. On 26-Jun-2008 we caught this female during the daytime in the males' roost and tagged her with a radio-transmitter. At dusk (20:00) Da left the males' roost with the males, and was recorded four hours later in the females’ roost 40 km to the south. Da spent 11 days in the females’ roost and foraged with the other females. On 8-Jul-2008 Da was recorded again in the males’ roost at midnight, and spent the day there. In the evening she was recorded with the males on the cliffs of the Naftali mountain range on the western side of the valley. The following night Da left the males' colony and was only recorded in the females’ foraging grounds during the night. On 17-Jul-2008 she returned to the females’ roost before sunrise and spent all the following days there until the end of the tracking period.

## Discussion

We found that both male and female *R. microphyllum* aggregate, and display complete sexual segregation during the summer in Israel, using different roosting and foraging sites. The two main hypotheses explaining such sexual segregation in vertebrates are those of reduced competition and/or different niche preferences. Roosting sites are known to be a limiting factor for bats using caves or tree cavities [35], [36]. We found many shallow dry caves and abandoned man-made structures which are suitable for the bats in this region, and therefore suggest that roosting sites are not limited for *R. microphyllum* in northern Israel. In Vespertilonid bats, males are usually smaller than females and it has been suggested that the males are displaced by the larger females to inferior regions [17]. We found no significant difference between the sexes in wing area or in body mass at the beginning of summer, when the bats inhabit the summer roosts. Moreover, no signs of aggression between males and females have ever been documented, even when an occasional male was spotted in the females' summer roost, or when both sexes were housed together in captivity (E. Levin, unpublished data). Therefore, we suggest that the sexes are not aggressively excluding one another from a preferred roosting site.

During summer, both sexes feed on a similar diet, composed mainly of winged ants, which could potentially result in competition for food resources [29]. However, we found that the two sexes forage at different sites (Figure 1), and as far as we could detect there is no difference in these foraging sites' quality. Both foraging sites abound with the main, high-quality food source – the alates of a very common carpenter ant, which perform nocturnal nuptial flights [29]. Both of these regions possess a greater abundance of food and water sources compared to most habitats in the arid distribution range of *R. microphyllum* [37]. The sharp increase in body mass and short foraging bouts during the summer months [31] further support our hypothesis that food is plentiful for both sexes during this season. Since food does not appear to be a limiting resource, it is reasonable to conclude that competition for food is not a factor driving sexual segregation in this species either.

Food may, however, be the driving force for aggregation in this species: as we show, *R. microphyllum* form large colonies, hunt in groups, and exploit an energy-rich and patchily distributed food source [29], [30]. As predicted by Safi and Kerth [25], they feed on patchy and swarming prey and have high aspect-ratio wings, and therefore will benefit from foraging in a group that will enable more efficient scanning of space and transfer of information via echolocation signals [26], [27]. It has been suggested that bats use their echolocation calls not only for spatial orientation and food acquisition, but also for information transfer regarding food source [38], [39], and individual recognition of their group members [39], [40], [41]. *R. microphyllum* use multi-harmonic CF echolocation calls. The frequency with maximum energy is located in the second harmonic (around 25 KHz), and the first harmonic is around 11 KHz [42] and can be well heard by the naked human ear. This relatively low frequency sound travels for relatively long distances in the atmosphere and can be used for communication between the bats.

The alternative hypothesis for the sexual segregation is that due to innate biological differences between the sexes they have different habitat preferences. Several biological and physiological differences between male and female *R. microphyllum* can lead to differences in roosting and foraging sites preferences and thus to sexual segregation. Roosting sites of males are located at higher altitudes than those of females. During summer, lactating *R. microphyllum* females remain normothermic while in the roosts, and accordingly perform longer foraging bouts than non-lactating females or males [31]. However, these different thermoregulatory strategies and energetic requirements, which could potentially result in different preferences for roosting sites with different ambient temperatures, do not appear to be the driving force for roosting site sexual segregation in this species, since we found no differences in the roosts' average temperatures or amplitude over summer [31].

Several differences between the sexes could potentially cause the foraging groups to be sexually segregated. Aerodynamic differences between sexes can affect the ability of the bats to establish synchronized group flight. Wing-load is an important factor affecting flight parameters, and differences in wing-load could greatly affect flight velocity and maneuverability in bats [43]. While both sexes of *R. microphyllum* have the same wing load when they arrive from the winter roosts, as the summer progresses this changes: the wing area of both sexes is equal and remains constant throughout the summer, but males gain body mass significantly more quickly and therefore their wing-load increases faster than that of the females. Consequently, it may be energetically favorable for bats not to fly in mixed groups of males and females during this period.

Another, not mutually exclusive, possible explanation for forming segregated foraging groups is related to energetic needs. During summer females are pregnant, give birth and lactate their young. Unlike males, lactating females do not enter torpor during this period [31]. However, both sexes need to accumulate sufficient fat during summer for the following hibernation period [30]. These differences probably lead to higher energetic requirements in reproductively active females, which result in longer foraging bouts compared to males [31]. Roosting in sexually segregated groups with similar energetic needs, and therefore similar foraging bout times, may allow energy–efficient, synchronized flying bouts, in which all members of the group leave and return to the roost together or in groups.

A major difference between the sexes was foraging habitat choice during the second phase of the nocturnal foraging (after the first hour of the forging bout). Both sexes spent the first hour after leaving the cave in low-lying regions with low elevation differences. Starting from the second hour of the night onwards, females maintained their preference for low elevation differences, while the males chose regions with high differences in elevation such as cliffs. Aerodynamically, males, which have higher wing-load during mid- and late summer, may prefer to forage around cliffs, where there are uplifting winds, while females remain in low- lying regions.

Foraging sites of the two sexes also differed in elevation, with males foraging in areas ca. 700 m higher than females, and in ambient temperatures that were much lower (Figure 1). It is possible that these differences in the foraging areas of the two sexes represent differences in their thermoregulatory requirements during activity. The hypothesis of activity-thermoregulatory heat substitution [44] suggests that heat produced during activity in the cold reduces the energy expenditure required for thermoregulation. The scope for heat substitution increases with an animal's body size and intensity of activity [44]. As T_a_ drops some of the heat produced by activity is used for thermoregulation, up to the point where heat produced by activity equals the amount needed for thermoregulation. Below this equilibrium point the animal has to start investing energy in thermoregulation. Bats invest much energy in flight, which is an energetically expensive form of locomotion [45]. Even though male and female *R. microphyllum* are similar in size, males accumulate significantly more fat during summer, and therefore are significantly heavier towards the end of summer. Moreover, males are expected to produce more heat during activity, due to their higher lean body mass [46]. Being heavier than the females, and producing more heat during activity, it is expected that T_a_ at the equilibrium point of heat substitution, which is optimal in terms of energy usage efficiency, will be lower for *R. microphyllum* males than females.

Other possible physiological, morphological and behavioral differences between the sexes (e.g., hormone levels, lactation, water balance, exercise capacity, adipose tissue to muscle ratio, body size, sociality, etc.) have the potential to influence ecological preferences [13], and affect behavioral and physiological aspects, such as aggregation in females [14], or differences in geographic dispersal patterns between the sexes [15]. It is possible that such biologically inate differences, as yet unexplored, between male and female *R. microphyllum,* also contribute to the sexual segregation during foraging. Based on the above findings, we suggest that in the subtropical *R. microphyllum* it is the optimization of energetic efficiency, by means of synchronized group foraging at the heat substitution equilibrium point, rather than competition, that is the main driver behind sexual segregation during summer. This aspect should be considered when discussing spatial sexual segregation in mammals and birds.

## Supporting Information

Table S1 **Number of tracking nights and positions taken for 38 bats during 2007–2008.**
(DOC)

Table S2 **Results of GWR models for the spatial dispersal of male and female bats in the first hour of foraging and afterwards.**
(DOC)
