# Supplementary material for: Bats of a Gender Flock Together: Sexual Segregation in a Subtropical Bat
Source: PLoS One. 2013 Feb 18;8(2):e54987. doi: 10.1371/journal.pone.0054987 (PMC3575394; doi:10.1371/journal.pone.0054987)
Supplement: Table S1 — Number of tracking nights and positions taken for 38 bats during 2007–2008. (DOC) [file pone.0054987.s001.doc]

**Table S1:**

| **Number of position collected** | **Number of nights tracked** | **Year** | **Sex** | **Bat code** |
| --- | --- | --- | --- | --- |
| 8 | 4 | 2007 | ♀ | 1 |
| 19 | 7 | 2007 | ♀ | 2 |
| 5 | 3 | 2007 | ♀ | 3 |
| 8 | 4 | 2007 | ♀ | 4 |
| 14 | 5 | 2007 | ♀ | 5 |
| 11 | 5 | 2007 | ♀ | 6 |
| 1 | 1 | 2007 | ♀ | 7 |
| 13 | 6 | 2007 | ♀ | 8 |
| 15 | 7 | 2007 | ♀ | 9 |
| 24 | 8 | 2008 | ♀ | Av |
| 14 | 4 | 2008 | ♀ | Or |
| 17 | 8 | 2008 | ♀ | Ga |
| 18 | 7 | 2008 | ♀ | Da |
| 14 | 5 | 2008 | ♀ | Ta |
| 2 | 2 | 2008 | ♀ | Yf |
| 23 | 8 | 2008 | ♀ | Le |
| 10 | 4 | 2008 | ♀ | Mi |
| 8 | 3 | 2008 | ♀ | Ma |
| 4 | 2 | 2008 | ♀ | Of |
| 11 | 5 | 2007 | ♂ | 10 |
| 2 | 1 | 2007 | ♂ | 11 |
| 8 | 2 | 2007 | ♂ | 12 |
| 11 | 5 | 2007 | ♂ | 13 |
| 9 | 4 | 2007 | ♂ | 14 |
| 3 | 2 | 2007 | ♂ | 16 |
| 10 | 5 | 2007 | ♂ | 17 |
| 8 | 3 | 2007 | ♂ | 18 |
| 10 | 4 | 2007 | ♂ | 19 |
| 22 | 6 | 2008 | ♂ | Aa |
| 5 | 2 | 2008 | ♂ | Ur |
| 5 | 1 | 2008 | ♂ | Be |
| 16 | 6 | 2008 | ♂ | Do |
| 4 | 1 | 2008 | ♂ | Io |
| 16 | 7 | 2008 | ♂ | Yo |
| 13 | 3 | 2008 | ♂ | Ni |
| 9 | 2 | 2008 | ♂ | Am |
| 24 | 7 | 2008 | ♂ | Zo |
| 4 | 1 | 2008 | ♂ | Sh |
