# Supplementary material for: Bats of a Gender Flock Together: Sexual Segregation in a Subtropical Bat
Source: PLoS One. 2013 Feb 18;8(2):e54987. doi: 10.1371/journal.pone.0054987 (PMC3575394; doi:10.1371/journal.pone.0054987)
Supplement: Table S2 — Results of GWR models for the spatial dispersal of male and female bats in the first hour of foraging and afterwards. (DOC) [file pone.0054987.s002.doc]

**Table S2:**

**Males: first hour GWR results**

| **Model parameters** | **K** | **AICc** | **R^2^** | **ΔAICc** |  | **W** |
| --- | --- | --- | --- | --- | --- | --- |
| Water resources | 2 | 76 | 0.32 | 0 | ≈1 | 1 |
| Settlements+ Water resources + Habitat | 4 | 156 | 0.25 | 79 | <0.0001 | 0.00 |
| Settlements+ Water resources + Habitat + Altitude SD | 5 | 171 | 0.24 | 94 | <0.0001 | 0.00 |
| Altitude SD | 2 | 187 | 0.25 | 111 | <0.0001 | 0.00 |
| Δ-Altitude | 2 | 188 | 0.25 | 111 | <0.0001 | 0.00 |
| Habitat | 2 | 215 | 0.17 | 138 | <0.0001 | 0.00 |
| Settlements | 2 | 216 | 0.16 | 139 | <0.0001 | 0.00 |
| Annual precipitation+ Water resources | 3 | 218 | 0.12 | 141 | <0.0001 | 0.00 |
| Maximum geographic altitude | 2 | 225 | 0.12 | 148 | <0.0001 | 0.00 |
| Habitat+ Altitude SD | 3 | 231 | 0.17 | 154 | <0.0001 | 0.00 |
| Settlements+ Water resources + Habitat + Annual precipitation | 5 | 233 | 0.11 | 156 | <0.0001 | 0.00 |
| Annual precipitation + Water resources + Altitude SD | 4 | 235 | 0.11 | 158 | <0.0001 | 0.00 |
| Minimum geographic altitude | 2 | 244 | 0.10 | 167 | <0.0001 | 0.00 |
| Settlements+ Water resources + Habitat + Annual precipitation | 5 | 247 | 0.10 | 171 | <0.0001 | 0.00 |
| Annual precipitation | 2 | 267 | 0.07 | 191 | <0.0001 | 0.00 |
| Annual precipitation + Altitude SD | 3 | 283 | 0.06 | 206 | <0.0001 | 0.00 |
| Annual precipitation + Minimum geographic altitude | 4 | 295 | 0.04 | 218 | <0.0001 | 0.00 |
| Annual precipitation + Altitude SD + Settlements | 3 | 293 | 0.05 | 217 | <0.0001 | 0.00 |
| Annual precipitation + Altitude SD + Habitat | 4 | 297 | 0.04 | 220 | <0.0001 | 0.00 |
| Average temperature in August | 2 | 306 | 0.02 | 229 | <0.0001 | 0.00 |

**Males: after the first hour GWR results**

| **Model parameters** | **K** | **AICc** | **R^2^** | **ΔAICc** |  | **W** |
| --- | --- | --- | --- | --- | --- | --- |
| Altitude SD | 2 | 2462 | 0.25 | 0.0 | ≈1 | 1.00 |
| Δ-elevation | 2 | 2482 | 0.24 | 20.4 | <0.0001 | 0.00 |
| Habitat + Altitude SD | 3 | 2488 | 0.21 | 26.5 | <0.0001 | 0.00 |
| Annual precipitation+ Altitude SD | 3 | 2490 | 0.20 | 28.1 | <0.0001 | 0.00 |
| Settlements+ Habitat+ Water resources+ Altitude SD | 5 | 2496 | 0.21 | 34.3 | <0.0001 | 0.00 |
| Annual precipitation+ Water resources+ Altitude SD | 4 | 2510 | 0.18 | 48.6 | <0.0001 | 0.00 |
| Annual precipitation+ Settlements + Altitude SD | 4 | 2512 | 0.18 | 50.6 | <0.0001 | 0.00 |
| Annual precipitation+ Habitat + Altitude SD | 4 | 2544 | 0.15 | 82.1 | <0.0001 | 0.00 |
| Settlements+ Water resources+ Altitude SD+ Annual precipitation+ Habitat | 7 | 2563 | 0.14 | 101.4 | <0.0001 | 0.00 |
| Minimum geographic altitude | 2 | 2594 | 0.11 | 131.8 | <0.0001 | 0.00 |
| Water resources | 2 | 2607 | 0.13 | 145.1 | <0.0001 | 0.00 |
| Maximum geographic altitude | 2 | 2623 | 0.08 | 160.8 | <0.0001 | 0.00 |
| Settlements | 2 | 2634 | 0.08 | 172.1 | <0.0001 | 0.00 |
| Habitat | 2 | 2634 | 0.08 | 172.2 | <0.0001 | 0.00 |
| Annual precipitation | 2 | 2641 | 0.06 | 179.4 | <0.0001 | 0.00 |
| Annual precipitation + Water resources | 3 | 2652 | 0.05 | 190.4 | <0.0001 | 0.00 |
| Settlements+ Habitat+ Water resources | 4 | 2656 | 0.05 | 193.9 | <0.0001 | 0.00 |
| Annual precipitation + Minimum geographic altitude | 3 | 2662 | 0.04 | 200.4 | <0.0001 | 0.00 |
| Annual precipitation + Settlements+ Habitat+ Water resources | 5 | 2671 | 0.03 | 209.1 | <0.0001 | 0.00 |
| Average temperature in August | 2 | 2682 | 0.02 | 220.6 | <0.0001 | 0.00 |

**Females: first hour GWR results**

| **Model parameters** | **K** | **AICc** | **R^2^** | **ΔAICc** |  | **W** |
| --- | --- | --- | --- | --- | --- | --- |
| Δ-Altitude | 2 | 491.1 | 0.30 | 0.0 | 1 | 0.99 |
| Altitude SD | 2 | 500.0 | 0.29 | 8.9 | 0.011765 | 0.01 |
| Water resource | 2 | 505.0 | 0.25 | 14.0 | 0.000934 | 0.00 |
| Altitude SD + Water resource | 3 | 511.4 | 0.25 | 20.3 | <0.0001 | 0.00 |
| Δ-Altitude+ Water resource | 3 | 511.5 | 0.25 | 20.4 | <0.0001 | 0.00 |
| Δ-Altitude+ Altitude SD+ Water resource | 4 | 519.1 | 0.24 | 28.0 | <0.0001 | 0.00 |
| Δ-Altitude+ Settlement+ Wetland+ Water resource +Habitat | 6 | 523.6 | 0.24 | 32.5 | <0.0001 | 0.00 |
| Annual precipitation + Water resource+ Δ-Altitude | 4 | 524.1 | 0.23 | 33.0 | <0.0001 | 0.00 |
| Settlement +Water resource +Wetland +Habitat | 5 | 535.4 | 0.23 | 44.3 | <0.0001 | 0.00 |
| Δ-Altitude + Habitat | 3 | 545.5 | 0.22 | 54.4 | <0.0001 | 0.00 |
| Settlement | 2 | 554.2 | 0.21 | 63.1 | <0.0001 | 0.00 |
| Habitat | 2 | 554.3 | 0.20 | 63.2 | <0.0001 | 0.00 |
| Settlement +Rater resource +Habitat | 4 | 579.0 | 0.18 | 87.9 | <0.0001 | 0.00 |
| Water resource | 2 | 596.1 | 0.14 | 105.1 | <0.0001 | 0.00 |
| Annual precipitation | 2 | 623.5 | 0.10 | 132.4 | <0.0001 | 0.00 |
| Annual precipitation+ Water resource | 3 | 627.1 | 0.09 | 136.1 | <0.0001 | 0.00 |
| Δ-Altitude+ Annual precipitation | 3 | 651.9 | 0.06 | 160.9 | <0.0001 | 0.00 |
| Annual precipitation + Δ-Altitude + Settlement | 4 | 655.4 | 0.05 | 164.3 | <0.0001 | 0.00 |

**Females: after first hour GWR results**

| **Model parameters** | **K** | **AICc** | **R^2^** | **ΔAICc** |  | **W** |
| --- | --- | --- | --- | --- | --- | --- |
| Δ-Altitude | 2 | 1747.5 | 0.36 | 0.0 | 1 | 0.82 |
| Altitude SD | 2 | 1750.6 | 0.36 | 3.0 | 0.218446 | 0.18 |
| Settlement | 2 | 1785.2 | 0.30 | 37.6 | <0.0001 | 0.00 |
| Wetland+ Δ-Altitude | 3 | 1809.8 | 0.26 | 62.3 | <0.0001 | 0.00 |
| Wetland | 2 | 1812.6 | 0.26 | 65.1 | <0.0001 | 0.00 |
| Wetland+ Altitude SD | 3 | 1815.2 | 0.26 | 67.6 | <0.0001 | 0.00 |
| Habitat | 2 | 1815.7 | 0.26 | 68.1 | <0.0001 | 0.00 |
| Settlement +Wetland+ Δ-Altitude | 4 | 1816.7 | 0.26 | 69.1 | <0.0001 | 0.00 |
| Δ-Altitude+ Habitat | 3 | 1818.1 | 0.27 | 70.6 | <0.0001 | 0.00 |
| Settlement +Water resource +Wetland+ Δ-Altitude+ Habitat | 6 | 1824.0 | 0.26 | 76.4 | <0.0001 | 0.00 |
| Settlement + Water resource +Wetland+ Habitat | 5 | 1831.1 | 0.25 | 83.6 | <0.0001 | 0.00 |
| Water resource | 2 | 1834.9 | 0.24 | 87.4 | <0.0001 | 0.00 |
| Annual precipitation + Wetland+ Δ-Altitude | 3 | 1835.8 | 0.23 | 88.3 | <0.0001 | 0.00 |
| Settlement + Water resource +Habitat | 4 | 1847.9 | 0.23 | 100.4 | <0.0001 | 0.00 |
| Annual precipitation | 2 | 1896.4 | 0.15 | 148.9 | <0.0001 | 0.00 |
| Annual precipitation +Water resource | 3 | 1901.8 | 0.15 | 154.2 | <0.0001 | 0.00 |
| Annual precipitation + Δ-Altitude | 3 | 1940.1 | 0.09 | 192.5 | <0.0001 | 0.00 |
| Annual precipitation+ Altitude SD+ Settlement | 4 | 1943.1 | 0.09 | 195.6 | <0.0001 | 0.00 |
